# Novel Cost-Effective and Portable Three-Dimensional Force Measurement System for Biomechanical Analysis: A Reliability and Validity Study

**DOI:** 10.3390/s24247972

**Published:** 2024-12-13

**Authors:** Letian Hao, Chao Yin, Xiaozhe Duan, Zeyu Wang, Meizhen Zhang

**Affiliations:** College of Physical Education and Health Engineering, Taiyuan University of Technology, Jinzhong 030600, China; hletian1022@163.com (L.H.); 18234507602@163.com (C.Y.); 13233419446@163.com (X.D.); 18135146256@163.com (Z.W.)

**Keywords:** force plate, ground reaction force, sensors, validation testing

## Abstract

The application of dynamic data in biomechanics is crucial; traditional laboratory-level force measurement systems are precise, but they are costly and limited to fixed environments. To address these limitations, empirical evidence supports the widespread adoption of portable force-measuring platforms, with recommendations for their ongoing development and enhancement. Taiyuan University of Technology has collaborated with KunWei Sports Technology Co., Ltd. to develop a portable 3D force measurement system. To validate the reliability of this equipment, 15 male collegiate students were randomly selected to perform four distinct movements: walking, running, CMJ, and side-cutting. The Bertec system served as a reference device alongside the KunWei system to collect the kinetic characteristics of the test movements. The consistency and fitting quality between the two devices were evaluated through *t*-tests, ICC, and NRMSE. The research results indicated that there were no significant differences in peak force between the KunWei system and the Bertec system across all four movements (*p* > 0.05). The ICC values for force-time curves were all above 0.98, with NRMSE not exceeding 0.165. The KunWei system exhibited high consistency and reliability under various motion conditions compared to the Bertec system. This system maintains data accuracy, significantly broadens the application scope of force measurement systems, and reduces procurement and maintenance costs. It has been successfully applied in technical support for multiple water sports and winter projects with ideal results achieved.

## 1. Introduction

In the realm of biomechanics, dynamic data are of paramount importance [1], with ground reaction force (GRF) emerging as a vital element. The GRF is crucial for sports training [2], injury prevention [3], and rehabilitation [4]. Prior studies have focused on GRF’s impact across a range of forces, ranging from low to high, and from unidirectional to multidirectional, as well as varying movement velocities [5]. Honert’s pivotal work on optimizing running techniques through GRF analysis has demonstrated a significant enhancement in athletic performance by reducing excessive GRF [6]. Additionally, McKeon’s use of GRF analysis to evaluate balance and stability changes in individuals with ankle sprains has been crucial in designing targeted rehabilitation strategies [7,8]. This approach has facilitated more effective recovery by adjusting landing patterns and redistributing power. These collective studies highlight the critical importance of GRF in both research and clinical settings [9]. Consequently, there is a pressing need for three-dimensional force measuring equipment that is not only highly accurate but also adaptable and cost-effective, capable of accommodating diverse motion modes. This equipment is essential for researchers and coaches, enabling efficient motion data collection and analysis across various movement scenarios [10,11]. It enhances the ability to optimize training and rehabilitation programs, advancing sports science and clinical practice.

A wide range of three-dimensional force measurement equipment is available [12], spanning from the esteemed laboratory-grade plates to the state-of-the-art wearable force-measuring devices [13]. Leading companies like Bertec, Kistler, AMTI, and Hawkins Dynamics, have set standards of excellence in ground reaction force measurement and data collection. These instruments are praised for their exceptional precision in measurement and the depth of data they provide, which is invaluable for comprehensive biomechanical assessments. Despite their technical sophistication, these devices face practical challenges that restrict their use in specific contexts [11]. Rebelo’s research underscores the inherent limitations of traditional force platforms, which are confined to controlled laboratory environments. This restriction limits their ability to collect motion data in real-world settings [14], hindering the capture of dynamic biomechanical performance in various training and competitive scenarios. Real-time data capture across multiple facilities is crucial for understanding biomechanical dynamics, a need unmet by current fixed-force platforms. Moreover, the financial implications of procuring such equipment are substantial [15]. The cost of state-of-the-art devices from leading manufacturers, such as Kistler and Bertec, can cost tens to hundreds of thousands of dollars, often exceeding the budgets of smaller research institutions and sports teams. The high cost of acquisition presents a formidable barrier to the collection of a broad and diverse dataset. These limitations highlight the urgent demand for a cost-effective, portable three-dimensional force platform that can adapt to diverse environments while maintaining accuracy and reliability in capturing biomechanical data.

To overcome the limitations of traditional force measuring tables, the collaboration between Taiyuan University of Technology and Kunwei Sports Technology Co., Ltd. has yielded the KunWei Mobile Force Measurement Instrument, a device designed to transcend the constraints of single-application scenarios and high costs. The system comprises three core components: mobile force plates, auxiliary platforms, and a motion analysis system. Its portability, adaptability to diverse testing environments, and competitive cost make it a strong contender in force measurement technology. To assess the stability and accuracy of the KunWei system across various movements, this study compared GRF data collected by the KunWei device with data from the Bertec standard commercial force measurement instrument. The aim was to verify the reliability of the KunWei system. This research aims to (1) verify the reliability and validity of the portable three-Axis force platform (KunWei system) in multiple motion modes; (2) compare the consistency of force data acquisition between the portable system and a commercial platform; (3) discuss the potential advantages and limitations of the portable force platform in practical applications. Based on the findings of previous studies, this investigation hypothesizes that there will be no significant statistical difference in peak forces, across all axes, for the four tested movements when using the KunWei device as opposed to the Bertec system. It is also hypothesized that the intraclass correlation coefficient (ICC) for the rhythmic force trajectories of the axial forces, as measured by the KunWei system, will show a high correlation with those measured by the Bertec system, with an ICC value greater than 0.95. Furthermore, the study posited that the root mean square error (RMSE) for the comparison of these force measurements will be below the threshold of 0.20, signifying strong agreement between the two systems.

### 1.1. Structural Composition and Innovative Features of the KunWei Force Plate System

**Figure 1:**
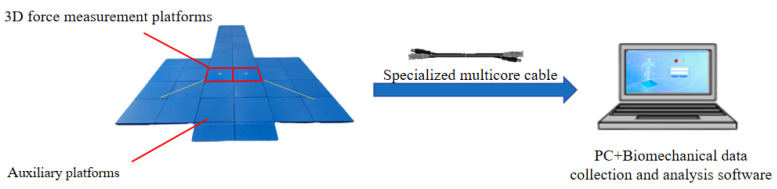
Constitution of the KunWei force platform system.

The KunWei system (KWYB-FP6050) is a sophisticated assembly that integrates a 3D force measurement platform, dedicated connecting cables, and specialized biomechanical data acquisition and analysis software (Figure 1).

The platform’s central structure, housed within a protective shell, is carefully designed to balance durability and stability. Made from high-strength, lightweight alloy plates, the main body is intricately integrated with four sets of state-of-the-art six-Axis force sensors. These sensors are strategically embedded at the corners beneath the main body, contributing to a construction that is both rigid and lightweight. This innovative layout supports a wide range of sampling frequencies for ground reaction force (GRF) testing, enhancing the system’s adaptability across diverse experimental settings. When connected to a user-supplied computer, the KunWei system demonstrates remarkable efficiency in the collection and processing of GRF data. The parameters and components of the KunWei system can be seen in Table 1 and Table 2.

The KunWei system precisely analyzes and processes sensor signal data, adeptly translating it into raw force measurements. The algorithmic procedure is outlined as follows:

Calculate the force output of the plate:

(1) Fx=X1+X2+X3+X4

(2) Fy=Y1+Y2+Y3+Y4

(3) Fz=Z1+Z2+Z3+Z4

Calculate the moment output of the plate:

(4) Mx=(Z1+Z4−Z3−Z2)∗Dy

(5) My=(Z4+Z3−Z2−Z1)∗Dx

(6) Mz=(X1+X4−X2−X3)∗Dy+(Y3+Y4−Y1−Y2)∗Dx

(Reference distance: *Dx* = 0.22/2 = 0.11 m, *Dy* = 0.12/2 = 0.06 m).

### 1.2. Definition of Coordinate System

**Figure 2:**
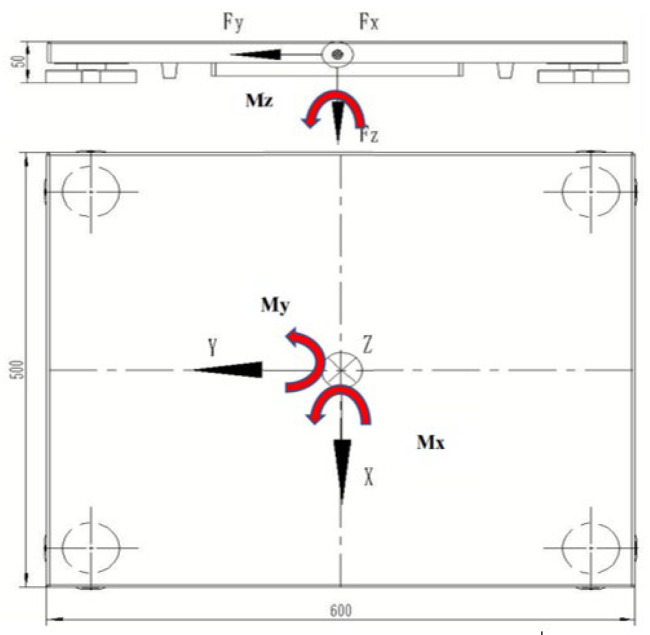
Defines the coordinate system of the force platform.

In this context, Fx, Fy, and Fz denote the respective components of the three-dimensional reactive forces, corresponding to the X, Y, and Z axes. Concurrently, Mx, My, and Mz symbolize the moments of these forces in three dimensions, each aligned with the respective axes. The orientation of these force moments is governed by the right-hand rule (Figure 2).

## 2. Materials and Methods

### 2.1. Participants

This study employed a paired sample *t*-test design. The test parameters were determined using G*Power 3.1.9.2 software with the following settings: the mean difference between two dependent means (matched pairs), effect size = 0.6, significance level (α) = 0.05, and statistical power (1 − β) = 0.8. The calculated required sample size was 24, with a minimum of 12 subjects needed after accounting for repeated measures. To ensure sufficient data, 15 male college students (height: 178.8 ± 11.2 cm; weight: 74.9 ± 8.7 kg; age: 20.4 ± 1.4 years) voluntarily participated in this study. Participants were screened to confirm no history of musculoskeletal injuries in the past six months. This criterion was strictly applied to maintain the accuracy of biomechanical measurements and ensure that participants could perform test movements without limitations or compensatory patterns.

### 2.2. Experimental Design

**Figure 3:**
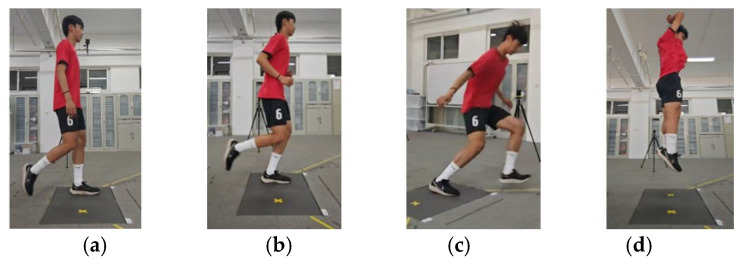
Test action diagram: (**a**) walking, (**b**) running, (**c**) side-cutting, and (**d**) CMJ.

The participants were outfitted with testing shoes and form-fitting attire provided by the laboratory. After a standardized warm-up and practice of the test actions, the experimental trials began. Participants performed four distinct biomechanical assessments, walking, running, side-cutting, and CMJ, in an order determined by a random number generator to ensure experimental rigor [16,17] (Figure 3). In walking and running trials, participants were instructed to move at a self-selected, comfortable pace, ensuring a natural gait and uniform velocity across trials. The left foot was required to contact the force plate. For the side-cutting maneuver, participants approached at a comfortable pace, landed on the force plate’s center marker with their right foot, and quickly accelerated leftward, ensuring their left foot contacted the guidance line. The central region of the force plate was marked with yellow tape to improve participants’ recognition during the assessment. The guidance line was positioned at a 45-degree angle to the initial running direction, ensuring that participants adhered to the correct trajectory throughout the test. During practice trials, participants were required to establish their starting position, and subsequently, they were instructed to concentrate on performing the movements with consistent technique, maintaining a forward gaze throughout the exercise. For the CMJ, participants were required to squat to their individual optimal depth at a self-selected pace before exerting a forceful vertical jump with both feet while using arm swings to maximize the jump height. Maintaining full leg extension was essential during the takeoff phase. During landing, participants returned both feet to the platform, prioritizing stability.

Comprehensive kinematic and kinetic data were collected using advanced KunWei force plates (Kunwei Sports Technology Corporation, China) and Bertec force plates (Bertec Corporation, USA), both operating at a high sampling frequency of 1000 Hz. This frequency ensured the accuracy and reliability of the data by capturing the nuances of each movement with precision. Each participant executed each movement a total of five times to ensure the collection of robust and valid datasets, thereby enhancing the statistical power and the generalizability of the findings.

### 2.3. Collection and Processing

In assessing the performance of our KunWei system, we conducted a comprehensive set of four exercise experiments. For comparative purposes, we respectively utilized the Bertec system to evaluate the accuracy of motion capture data. Our experimental setup included two cutting-edge three-dimensional force platforms manufactured by Bertec. These platforms were synchronized to operate at a uniform sampling rate of 1000 Hz, thereby facilitating a robust and reliable comparative analysis.

The original kinematic data from Bertec was processed and exported using Cortex-642.6.2 software (Motion Analysis, Rohnert Park, CA, USA), while the original kinematic data from KunWei was processed and exported using KunWei Motion Technology biomechanical data collection and analysis software (KunWei Motion Technology, Shanghai, China). According to the criteria, a vertical GRF greater than 10 N indicates the start, while less than 10 N indicates the end [18]. Considering the distinct directional distribution of certain kinetic forces and the lack of identifiable peaks along these axes, we have focused our analysis on the Y-Axis and Z-Axis data during running and walking tests. For the CMJ tests, we have exclusively analyzed the Z-Axis data. In contrast, during the transverse cutting motion tests, we have included data from all three axes (X, Y, Z) in our analysis.

### 2.4. Statistical Analysis

Dynamics data were normalized relative to the athlete’s body weight, expressed as the ratio of dynamic parameters to body weight. The Shapiro–Wilk test was applied to each variable to verify the normality of the data distribution. The results confirmed that all experimental data adhered to a normal distribution. Therefore, parametric tests were considered suitable for subsequent analyses. All statistical computations were conducted using SPSS version 27 (SPSS Inc., Chicago, IL, USA). Specifically, paired *t*-tests were utilized to compare the peak mean values recorded by two force plates. The threshold for statistical significance was set at a level of 0.05, ensuring that the probability of a Type I error did not exceed this value. The coefficient of variation (CV) was calculated as the ratio of the standard deviation to the mean, expressed as a percentage, to assess the relative variability of the data. Moreover, a two-way random effects model was employed to estimate the ICC for the two datasets. The ICC is a critical metric for evaluating the consistency or reliability of different measurement techniques when applied to identical quantitative outcomes [19]. According to the established criteria, data consistency is considered poor if the ICC value lies between 0 and 0.40, and good if it ranges from 0.75 to 1.00 [20]. The specific methodology for calculating the ICC is delineated in the subsequent equation [21]:

(7) $$\mathrm{ICC}=\frac{\mathrm{MSB}-\mathrm{MSW}}{\mathrm{MSB}+\left(k-1\right)\times \mathrm{MSW}+\frac{k\times (\mathrm{MSE}-\mathrm{MSW})}{n}}$$

MSB: Mean Square Between
MSW: Mean Square Within
k: The number of samples in each group
MSE: Mean Square Error
n: Total number of samples.

The quality of fitting for the periodic curves generated by the two types of force plates was rigorously evaluated through the computation of the normalization root mean square error (*NRMSE*). This metric, widely recognized in the scholarly literature, was calculated by dividing the *RMSE* by the range of the observed data, yielding a value within the [0,1] interval. An *NRMSE* threshold not exceeding 0.2 is generally accepted as indicative of a commendable fit within the control group, thereby providing a robust benchmark for assessing the accuracy of the force plate data [22,23]. The specific methodology for calculating the *RMSE* is delineated in the subsequent equation [24]:

(8) $$\mathrm{RMSE}=\sqrt{\frac{1}{n}\sum_{i=1}^{n}(y_{i}-\hat{y_{i}}{)}^{2}}$$

The *NRMSE* uses Min–MAX normalization method. The specific method of normalization is described in the following equation [25]:

(9) $$\mathrm{NRMSE}=\frac{\mathrm{RMSE}}{y_{\max}-y_{\min}}$$

### 2.5. Ethics Statement

The studies involving human participants were reviewed and approved by the Taiyuan University of Technology ethics committee (Ethical Approval Number: TYUT2024061901) and adhered to the principles of the Declaration of Helsinki. Prior to their participation, all participants were duly informed about the study and provided their voluntary written consent.

## 3. Result

### 3.1. Peak Force t-Test

The *t*-test analysis has revealed a significant correlation in peak forces between the KunWei and Bertec force plates across a variety of conditions. During the walking trials, the Y-Axis forces (*t* = −1.500, *p* = 0.138) and the primary (*t* = 0.712, *p* = 0.479) and secondary (*t* = 0.351, *p* = 0.351) Z-Axis force peaks did not exhibit statistically significant differences, suggesting a high degree of agreement between the two force plate systems. This consistency was also observed during the running phase, where no significant discrepancies were noted in the Y-Axis (*t* = −1.508, *p* = 0.136) or Z-Axis (*t* = 1.218, *p* = 0.227) forces. In the side-cutting maneuvers, while the Z-Axis forces (*t* = 0.150, *p* = 0.881) were not significantly divergent, there were substantial and statistically significant differences in the X-Axis (*t* = 4.625, *p* < 0.001) and Y-Axis (*t* = −3.928, *p* < 0.001) forces. For the CMJ, there was no statistically significant difference in the Z-Axis forces measured by the KunWei and Bertec force plates (*t* = −0.787, *p* = 0.277), further confirming the reliability of both devices under this specific dynamic condition. Except for the X- and Y-axis during side-cutting maneuvers, the 95% confidence interval (CI) for the difference in peak forces across the four actions, as measured by both devices, included zero. This finding indicates a high level of consistency in their measurement outcomes under these conditions. In contrast, the 95% CI for the difference in the Side-cutting X-Axis was [0.077, 0.194], and for the Side-cutting Y-Axis was [−0.235, −0.077], neither of which included zero. The coefficient of variation (CV) values across various actions indicate comparable stability between the two systems. Additionally, the Pearson correlation coefficients (r) show significant positive correlations across most actions (r > 0.8). Regression analysis further validates the CV values, revealing B values close to one across various movement patterns (walking Z-Axis B = 1.059; running Z-Axis B = 0.741; CMJ Z-Axis B = 0.895). This indicates a strong agreement between measurements from the KunWei system and the Bertec system. A comparison of some of the *t*-test results of each force plate can be seen in Table 3.

**Figure 4:**
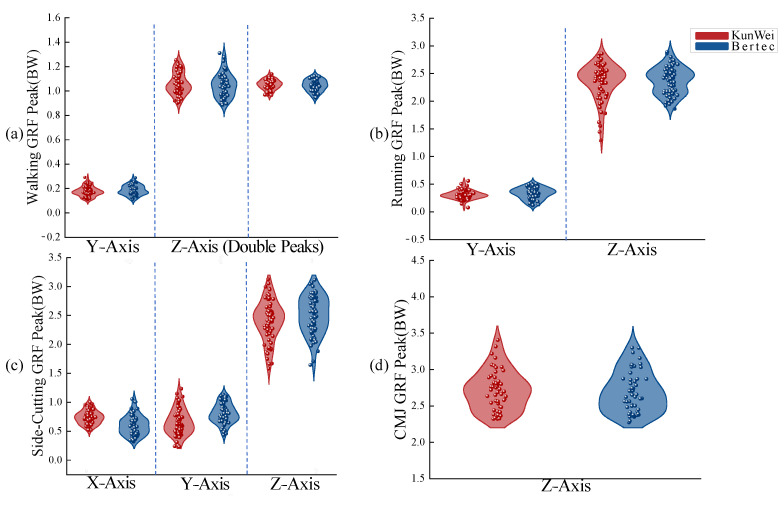
Four actions GRF peaks distribution for the KunWei and the Bertec system. (**a**) Walking GRF Peak; (**b**) Running GRF Peak; (**c**) side-Cutting GRF Peak; (**d**) CMJ GRF Peak.

Although discrepancies in peak GRF were noted between the KunWei and Bertec force plates under certain conditions, the GRF peak distribution patterns were closely aligned in most movement scenarios, particularly in the Z-Axis distribution. This alignment indicated that the GRF data collected by two types of force plates exhibited a high validity across different types of actions (Figure 4).

**Figure 5:**
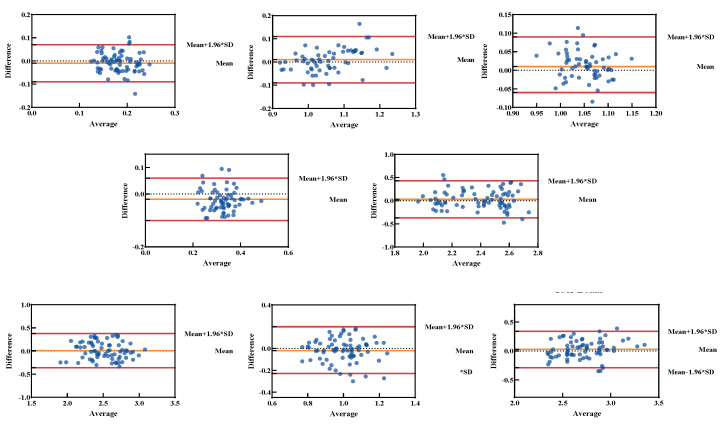
Bland–Altman plots of peak differences for the KunWei and Bertec systems. The blue dots on the plot represent the measurement results from a single subject obtained during one test. The X-axis represents the mean of measurements from the two force plates, the Y-axis represents the difference between the two instruments, and the center line indicates the mean bias.

The Bland–Altman analysis illustrates the agreement and variability in peak force measurements between the KunWei and Bertec systems across various testing conditions (Figure 5). In walking trials, the 95% limits of agreement (LOA) for peak force differences ranged from −67.62 N–78.65 N, reflecting a relatively narrow variability range. While running in the forward direction, the LOA ranged from −75.7 N–41.16 N, also demonstrating acceptable consistency. For dynamic and multidirectional actions, variability slightly increased. During side-cutting in the horizontal direction, the LOA ranged from −161.7 N–144.06 N. In the vertical direction, the LOA for running, side-cutting, and CMJ ranged from −271.95 N–316.06 N, −260.93 N–276.36 N, and 205.8 N–249.9 N, respectively. These wider LOA ranges indicate larger differences under conditions involving high peak forces (>1500 N).

### 3.2. Phases of the Movement Cycles ICC

**Figure 6:**
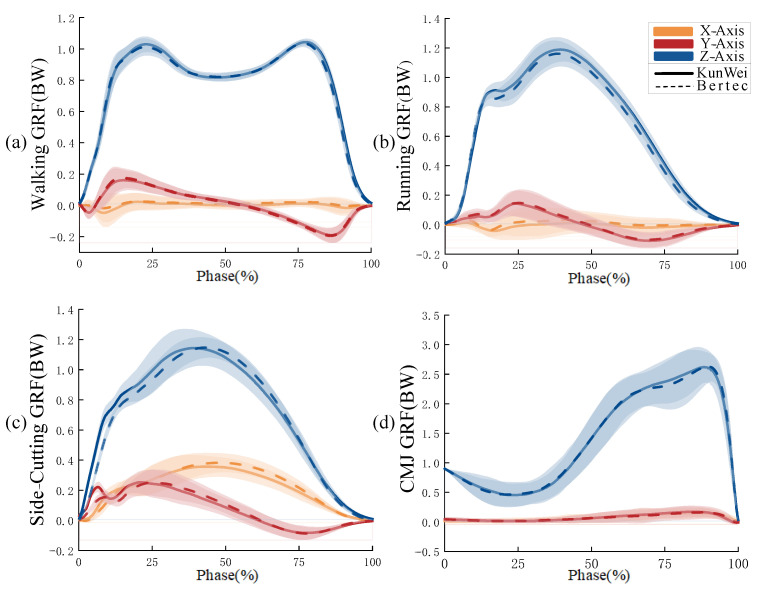
Four actions GRF phase for KunWei and Bertec system. (**a**) Walking GRF phase; (**b**) Running GRF phase; (**c**) side-Cutting GRF phase; (**d**) CMJ GRF phase.

The ICC results indicated a high level of consistency between the GRF curves obtained from the KunWei and Bertec force plates (Figure 6). In walking, the force plates exhibited a strong correlation in the X-Axis (*r* =0.950, *p* < 0.001), Y-Axis (*r* = 0.999, *p* < 0.001), and Z-Axis (*r* = 0.999, *p* < 0.001). During running, the consistency was similarly high, with the X-Axis showing (*r* = 0.912, *p* < 0.001), the Y-Axis showing (*r* = 0.999, *p* < 0.001), and the Z-Axis showing (*r* = 0.999, *p* < 0.001). For side-cutting, the force plates demonstrated a high degree of agreement in the X-Axis (*r* = 0.993, *p* < 0.001), Y-Axis (*r* = 0.988, *p* < 0.001), and Z-Axis (*r* = 0.996, *p* < 0.001). In the context of the CMJ, the two force plates showed a perfect agreement on the z-Axis (r = 0.999, *p* < 0.001).

In addition to the walking and running X-axes, the ICC values and the lower bounds of their respective 95% confidence intervals for the period curves on each Axis, as collected by the two force plates across the four movement types, consistently exceeded 0.950. Specifically, for the walking X-Axis, the ICC value and the lower limit of the 95% CI were 0.950 and 0.945, respectively. Similarly, for the running X-Axis, these figures were 0.912 and 0.892, respectively. A comparison of some of the ICC results of each force plate can be seen in Table 4.

### 3.3. Phases of the Movement Cycles NRMSE

**Figure 7:**
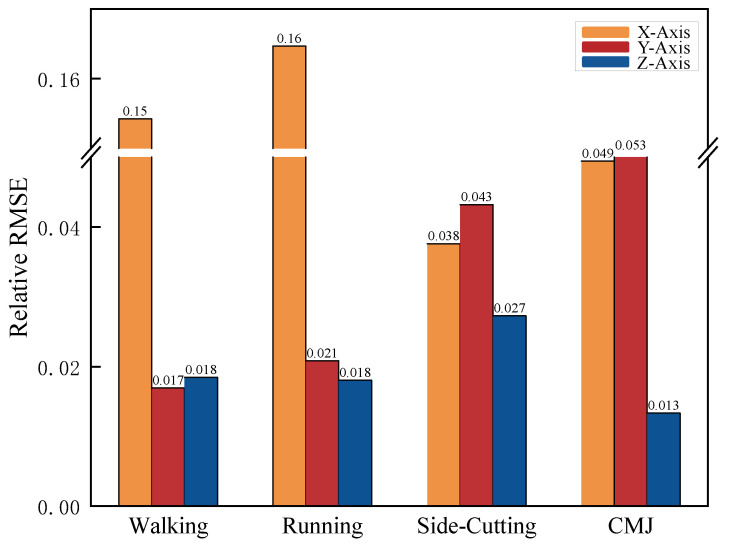
Four actions GRF curves relative NRMSE for the KunWei and the Bertec system.

The analysis of relative NRMSE indicated that the errors across different actions were consistently similar, despite minor discrepancies along each Axis (Figure 7). Notably, the X-Axis showed a higher NRMSE in most actions, including walking (0.154) and running (0.165). In contrast, the Z-Axis demonstrated a relatively lower error in most actions, CMJ (0.013). This indicated that the error difference between the two force plates is small when collecting different motion modes. Even in more complex and rapid actions, such as side-cutting, the error discrepancy did not significantly increase, and the overall error magnitude remained within a low threshold (NRMSE ≤ 0.1615).

## 4. Discussion

The results of our study substantially affirm the initial research hypothesis: when comparing the KunWei system with the Bertec system, no statistically significant differences in peak force measurements were observed across all axes for the four test movements, as ascertained by paired sample *t*-tests. The data revealed that the peak forces recorded during walking, running, CMJ, and lateral cutting movements did not exhibit significant disparities (*p* > 0.05) between the two force plate systems. Discrepancies noted in the X- and Y-axis during lateral cutting movements are hypothesized to stem from the variability inherent in repeated measurements on both force platforms, potentially introducing errors. This hypothesis aligns with prior research on the dynamics of side-cutting movements [26]. Butler’s [27] study posits that such variability and measurement errors may be attributable to the inconsistent angles of knee joint internal rotation and trunk inclination throughout the movement, which can vary trial by trial, thus amplifying measurement discrepancies. Upon further examination of the horizontal resultant forces on the X- and Y-axis during lateral cutting movements, no significant differences were identified (*p* = 0.539), as confirmed by paired sample *t*-tests [28], The comparison of *t*-test results can be seen in Table 5. Meanwhile, the coefficient of variation (CV) from peak testing, alongside Pearson correlation coefficients and regression analysis results, validated the consistency and reliability of the KunWei system compared to the Bertec system across various athletic scenarios. The CV values offered critical insights into variability across different movement types and repeated trials along each axis, highlighting the KunWei system’s superior measurement stability during dynamic and multidirectional motions. Pearson correlation coefficients (r > 0.6, with some values exceeding 0.8) indicated a high level of consistency in measurement outcomes across various axes and movement types between the two systems. Notably, for both CMJ and side-cutting actions, the Z-Axis correlation coefficient consistently exhibited high values, reflecting strong alignment in vertical force measurements between the two devices. This finding aligns with previous validation studies on force platforms, further supporting the reliability of the KunWei system. Regression analysis offered additional quantitative evidence of the relationship between the two systems, revealing robust linear consistency in peak force measurements. This analysis quantified their interrelationship and complemented findings from CVs and Pearson correlation coefficients. Additionally, high B values indicated that the KunWei system effectively replicates Bertec’s measurement trends with minimal deviation. The Bland–Altman analysis confirmed the consistency of two force measurement systems across various motion scenarios, with most data points within the LOA. While peak force deviation slightly increased, the error remained within practical limits. Consistent with Rebelo [14], who noted systematic biases in portable devices under complex conditions, this study reinforces their reliability and practical value for biomechanical analysis. Furthermore, The t-test results showed that the 95% confidence interval (CI) for differences in peak forces across the four movements, as measured by both force plates, included zero. This finding indicates a high degree of consistency in the measurements under the tested conditions [29]. The uniform distribution of GRF peak values across all axes for each movement mode underscores the KunWei system’s ability to deliver highly consistent GRF measurements in concert with the Bertec system across multiple trials. In the aggregate, these findings lend robust support to the reliability and validity of the KunWei system, suggesting its dependability as a tool for biomechanical analysis.

The results confirm the second hypothesis: the Intraclass ICC for the periodic force curves in each Axis, as measured by the KunWei system for the four test movements, were not less than 0.95 when compared to those collected by the Bertec system, and the NRMSE did not exceed 0.20. Specifically, ICC values for the periodic force curves on each Axis across the four test movements, collected by both platforms, exceeded 0.98, and NRMSE values were below 0.165. Furthermore, the 95% CI for the various actions and axial force periodic curves derived from both systems exhibited a narrow range [30], indicating high consistency and reliability. Carina’s study evaluated three foot-pressure measurement systems for ICC fit across varying pressure levels [31]. The research concluded that a mean ICC value of 0.638 or higher signifies good system performance. Another study, which assessed gait and lower limb motion using Inertial Measurement Units (IMUs), established an ICC value of no less than 0.750 as the benchmark for good system fit [32]. This research adopted a more stringent criterion, with all ICC values exceeding 0.900, and those on the Y-Axis and Z-Axis approaching one. This finding highlights the exceptional accuracy and reliability of the KunWei system, which meets even higher precision standards [33]. While the NRMSE values for the X-Axis during walking and running movements were relatively higher for both systems, they remained within an acceptable range. As the X-Axis represents a non-progression direction, values below 0.2 are widely recognized in the literature as acceptable for the control group [22,23]. This suggests that the periodic data collected by the KunWei system fits well when compared to the Bertec system [23,34]. Additionally, the GRF periodic curves for each Axis across the four movements from both systems showed a high degree of similarity, especially on the Z-Axis during walking, where the curves nearly overlapped, with dual peaks around 25% and 75% of the cycle [35]. Although the peak values on the X-Axis and Y-Axis are smaller, their trends and variations are highly similar between the two systems, demonstrating the KunWei system’s reliability despite variations in force magnitude or direction. These combined findings reinforce the reliability and validity of the KunWei system.

The KunWei system has been widely utilized in various competitive sports events for the Chinese national team, offering vital technological support to augment athletic performance. During preparations for the 2022 Beijing Winter Olympics and the 2020 Tokyo Olympics, the KunWei system, combined with wind tunnel experiments, provided critical kinematic data to the national skiing, rowing, and swimming teams, allowing real-time technique refinement. Its adaptability in various application scenarios, coupled with its reliability in data acquisition, positions the KunWei system as a versatile tool applicable to a broad range of sports, significantly enhancing the training efficiency and competitive performance of athletes. The practical applications of the KunWei system align with this study’s validation results, highlighting its ability to provide accurate kinematic data, broaden data collection scenarios, and reduce equipment costs.

In conclusion, the comparative analysis between the KunWei system and the Bertec system demonstrates a high degree of congruence in peak force measurements and curve fitting. The maximum force peak values across the four movement modes show no statistically significant differences on any axis, with a 95% CI encompassing zero. The ICC values for the force cyclic curves are all above 0.98, and the NRMSE values are all below 0.165, significantly surpassing the standard requirements of similar studies. The precision and trend of the GRF collected by the KunWei system in various axes and movement modes are notably similar, particularly in the Z-Axis, where the collection results are almost entirely consistent. This further substantiates the stability and reliability of the system. These research findings indicate that the data procured from the KunWei system are in alignment with the kinetic data collected by the Bertec system, suggesting its suitability for clinical kinetic data collection.

Ensuring data authenticity and reliability requires experimental tests to closely replicate real-life movement scenarios, moving beyond the limitations of a static laboratory environment [36]. Traditional force plate systems, like Bertec, are confined to laboratory use due to their immobility [37]. In this study, both force plate systems were evaluated in a controlled laboratory setting to minimize the influence of environmental variables on results. The experimental findings have confirmed the portable force plate system’s efficacy and stability in capturing kinematic data within a fixed laboratory context. The flexibility of the KunWei force plate system enables a more realistic setup for diverse sports testing scenarios. However, further investigation is needed to evaluate the system’s performance in complex, dynamic movement scenarios. Future studies will, thus, rigorously evaluate the KunWei system’s performance across a range of sports scenarios, providing robust empirical evidence to fuel the evolution and broader adoption of portable force plate systems. Looking ahead, the KunWei system aims to enhance data collection accuracy through the integration of sophisticated algorithms and increased automation. It will automatically detect human movement, collect kinematic data, and align with predefined motion models to extract key kinematic parameters. Additionally, the system aims to bolster its presentation of data and imagery, streamlining the analysis process. To improve portability, efforts will focus on reducing the system’s weight and enabling rapid deployment, aiming for precise kinematic data collection in complex and realistic sports settings. Strategic plans include expanding the system’s applications in sports and entering the medical rehabilitation sector, positioning it as a versatile tool for biomechanical research and clinical practice.

## 5. Conclusions

This study confirmed the high reliability and validity of the KunWei three-dimensional force measurement platform, with no significant differences in peak force measurements compared to the Bertec system. With ICC values exceeding 0.98 and RMSE below 0.165, the KunWei system demonstrated precision and consistency, establishing it as a cost-effective alternative to traditional laboratory-based force plates. Its portability, affordability, and adaptability enabled biomechanical analysis in diverse environments, including applications in sports training, rehabilitation, and clinical diagnostics. Successfully deployed in high-level athletic training, the KunWei system has demonstrated practicality and effectiveness. Future research will focus on enhancing automation and data processing to extend its applications to complex and dynamic movements. Continuous refinement will improve the system’s usability and impact, reinforcing its role in biomechanical research and practical applications, thereby contributing to advancements in sports science and clinical practice.

## Figures and Tables

**Table 1:** Specification of Kunwei force platform system.

Parameter	Rated Value	Nonlinearity	Sample Frequency
Force	XY: ±2500 [N] Z: ±5000 [N]	≤ ±0.2 [%FS]	50–1600 [Hz]
Moment	XYZ: ±950 [N·m]		

**Table 2:** Dimension of Kunwei force platform system.

Component	Width	Depth	Height	Weight
Measurement platform	500 [mm]	600 [mm]	50 [mm]	12 [kg]
Auxiliary platform			40–60 [mm]	8 [kg]

**Table 3:** Four actions GRF peaks in X, Y, and Z axes (mean ± standard). * Indicates that there are significant differences between the KunWei system and the Bertec system. ^1^ The 95% confidence interval for the difference. ^2^ The running action is studied in the vertical direction on the double peak.

Variables	KunWei		Bertec		*p* Value	95% CI (Difference) ^1^	Pearson r	B
	M ± SD (BW)	CV (%)	M ± SD (BW)	CV (%)				
Walking-Y	0.177 ± 0.037	20.9	0.186 ± 0.040	21.5	0.138	−0.020~0.003	0.373	0.330
Walking-Z	1.062 ± 0.090 ^2^ 1.052 ± 0.040	8.5 3.8	1.049 ± 0.098 1.045 ± 0.048	9.3 4.6	0.479 0.351	−0.023~1.049 −0.009~0.024	0.813 0.677	1.059 0.542
Running-Y	0.308 ± 0.078	25.3	0.330 ± 0.102	30.9	0.136	−0.500~0.007	0.759	0.741
Running-Z	2.400 ± 0.226	9.4	2.372 ± 0.237	10.0	0.227	−0.018~0.076	0.610	0.583
Side-cutting-X	0.738 ± 0.118 *	16.0	0.603 ± 0.172	28.5	<0.001	0.077~0.194	0.621	0.596
Side-cutting-Y	0.651 ± 0.223 *	34.3	0.806 ± 0.172	21.3	<0.001	−0.235~−0.077		
Side-cutting-Z	2.416 ± 0.340	14.1	2.404 ± 0.588	24.5	0.881	−0.156~0.181	0.760	0.870
CMJ-Z	2.717 ± 0.627	23.1	2.693 ± 0.259	9.6	0.277	−0.020~0.069	0.843	0.895

**Table 4:** ICC of the 4-action axial force period curve collected by the KunWei system and the Bertec system. ^1^ The 95% confidence interval.

Variables	X-Axis		Y-Axis		Z-Axis	
	*r*	95% CI ^1^	*r*	95% CI	r	95% CI
Walking	0.950	0.945~0.956	0.999	0.999~0.999	0.999	0.999~0.999
Running	0.912	0.892~0.927	0.999	0.999~0.999	0.999	0.999~0.999
Side-Cutting	0.993	0.992~0.994	0.998	0.985~0.990	0.996	0.995~0.996
CMJ	0.909	0.899~0.919	0.998	0.995~0.999	0.999	0.999~1.000

**Table 5:** Evaluation index of side-cutting horizontal force collected by the KunWei system and the Bertec system.

Variables	*T*-test			ICC		NRMSE
	KunWei (BW)	Bertec (BW)	*p* Value	*r*	95%CI	
Horizontal Force	0.972 ± 0.196	0.995 ± 0.181	0.539	0.986	0.983~0.988	0.047

## Data Availability

The data presented in this study is available on request from the corresponding author. The data are not publicly available for reasons of confidentiality.
